# Characterization of the Complete Mitochondrial Genomes from Two Nitidulid Pests with Phylogenetic Implications

**DOI:** 10.3390/insects11110779

**Published:** 2020-11-11

**Authors:** Xiaoxiao Chen, Qing Song, Min Huang

**Affiliations:** Key Laboratory of Plant Protection Resources and Pest Management of the Ministry of Education, Entomological Museum, College of Plant Protection, Northwest A&F University, Yangling 712100, Shaanxi, China; chenxiaoxiao@nwafu.edu.cn (X.C.); songqing@nwafu.edu.cn (Q.S.)

**Keywords:** Cucujoidea, mitochondrial genome, Nitidulidae, phylogeny

## Abstract

**Simple Summary:**

Nitidulidae is the most diverse group of Cucujoidea. In this study, we sequence and analyze two mitochondrial genomes of the nitidulid species, *Xenostrongylus variegatus* and *Epuraea* sp. Both genomes are highly conserved in their size, base content, codon usage, and secondary structure of tRNAs. Phylogenetic analyses of Nitidulidae and related families in Cucujoidea are carried out based on three outgroups and fourteen ingroups. The results of the Bayesian inference and maximum likelihood methods support the monophyly of Nitidulidae and the sister-group relationship of Kateretidae + (Monotomidae + Nitidulidae).

**Abstract:**

The complete mitochondrial genomes of *Xenostrongylus* *variegatus* and *Epuraea* sp. were sequenced and analyzed. The total genome lengths are 17,657 and 16,641 bp, with an A+T content of 77.2% and 76.4%, respectively. Each mitochondrial genome consists of 37 coding genes and a non-coding (AT-rich) region. All protein-coding genes (PCGs) start with the standard start codon, ATN, and end with complete stop codons, TAA and TAG, or an incomplete stop codon, T. All tRNAs can be folded into the typical clover-leaf secondary structure, with the exception of *trnS1* in both species with a reduced dihydrouridine (DHU) arm. The AT-rich region has tandem repeats differing in both number and length. Genetic distance and Ka/Ks analyses show that *nad6* has a higher variability and more rapid evolutionary rate than other PCGs. Both maximum likelihood and Bayesian inference phylogenetic analyses based on 13 PCGs and 2 ribosome DNAs (rDNAs) agree with the previous phylogenies in supporting the Nitidulidae monophyly and the sister-group relationship of Kateretidae + (Monotomidae + Nitidulidae).

## 1. Introduction

Nitidulidae is the largest group within the Cucujoidea (Coleoptera, Polyphaga), containing 350 genera in ten subfamilies, with nearly 4500 species worldwide [1,2]. Members of Nitidulidae inhabit a wide range of habitats in the Holarctic, Oriental, and Afrotopical Regions [3,4]. Many nitidulid species are pests of grain and other cash crops, seriously impacting plant pollination and seed production, and also spreading fungal pathogens [5,6,7,8,9,10,11,12,13,14,15,16,17,18]. *Xenostrongylus variegatus* Fairmaire, 1891, and *Epuraea* sp., the two species treated here, are also important pests of oilseed rape [19,20] and beehives respectively, with both widely distributed across China. 

Nearly all morphological and molecular data analyzed to date support the monophyly of Nitidulidae [1,21,22], except Tang’s analysis nesting Nitidulidae within Erotylidae based on mitochondrial genomes [23], and Bocak’s analysis nesting Passandridae within Nitidulidae [24]. However, Tang’s and Bocak’s analyses did not specifically focus on Nitidulidae and included very few species of Nitidulidae, so the results were not conclusive.

The phylogenetic relationship of Nitidulidae to other cucujoid families also remains unclear. Most morphological data support the sister relationship of Nitidulidae + Kateretidae [22,25,26,27], and this result is also supported by certain studies based on gene fragments, such as Cline et al. [21], based on seven loci (*12S*, *16S*, *18S*, *28S*, *COI*, *COII,* and *H3*), and Robertson et al. [2], based on eight loci (*18S*, *28S*, *H3*, *CAD*, *12S*, *16S*, *COI,* and *COII)*. The sister-group relationship of (Nitidulidae + Kateretidae) with Monotomidae was also supported by Bocak et al.’s [24] study based on four loci (*18S*, *28S*, *rrnL,* and *COI)*. Nevertheless, Hunt [28] suggested that Nitidulidae is closer to Monotomidae than to Kateretidae. Leschen noted that even though Nitidulidae and Monotomidae share an apparent morphological apomorphy, i.e., abdominal tergite VII exposed in dorsal view and tergite VIII in males with sides curved ventrally forming a genital capsule, their sister relationship is still doubtful [25]. So, further phylogenetic studies are needed in order to clarify the relationships between Nitidulidae and related families of Cucujoidea. 

So far, only five complete nitidulid mitochondrial genomes (*Epuraea guttata* (Olivier, 1811), *Carpophilus dimidiatus* (Fabricius, 1792), *Carpophilus pilosellus* (Motschulsky, 1858), *Aethina tumida* (Murray, 1867), and Nitidulidae sp.) have been published in GenBank. In this study, we present the mitochondrial genomes of two additional nitidulid species, *Xenostrongylus variegatus* and *Epuraea* sp., annotating and analyzing their structures in detail. We reconstruct the phylogenetic relationships of Nitidulidae and related families of Cucujoidea based on 13 protein-coding genes (PCGs) and 2 rRNAs of 17 taxa, including three outgroups and fourteen ingroups of insects. The purpose of this study is to improve our understanding on the mitochondrial characteristics of Nitidulidae and its phylogenetic relationships with related families.

## 2. Materials and Methods 

### 2.1. Materials and DNA Extraction

*Xenostrongylus variegatus* was collected from Xiaozhongdian, Shangri-La, Yunnan Province, China, in 2018. *Epuraea* sp. was collected from honeycomb in Xishuangbanna, Yunnan Province, China, in 2019. All materials were preserved in 100% ethanol and stored at −80 °C in the Entomological Museum of the Northwest A&F University. The total genomic DNA was extracted using the DNeasy DNA Extraction kit (Qiagen) after the morphological identification.

### 2.2. Sequence Analysis

The mitochondrial genomes of *X. variegatus* and *Epuraea*. sp. were sequenced by next-generation sequencing (NGS; Illumina HiSeq X10; 5.46 gb raw data; by Biomarker Technologies Corporation, Beijing, China). The raw data were preprocessed, then assembled and annotated with the default parameters used in the mitochondrial genomes of *C. dimidiatus* and *C. pilosellus* as the reference sequences, respectively. Default parameters were performed by Geneious 8.1.3 (Biomatters, Auckland, New Zealand) [29]. The 13 PCGs were identified by finding open reading frames (ORFs) and were translated into amino acids according to the invertebrate mitochondrial genetic code. The positions and secondary structures of 22 tRNAs were predicted by the MITOS Web Server (http://mitos.bioinf.uni-leipzig.de/index.py) [30]. Then, we manually edited the clover-leaf secondary structure with Adobe Illustrator CS5 according to the predicted structures. Two rRNAs and the AT-rich region were identified by the location of adjacent genes and through comparison with other reported homologous sequences of members of Nitidulidae. Mitogenomic circular maps were produced using CGView Server (http://stothard.afns.ualberta.ca/cgview_server/) [31]. The base composition, component skew, and codon usage of the PCGs and relative synonymous codon usage (RSCU) were analyzed using PhyloSuite v1.2.1 [32]. Tandem repeats of the control region were established by the Tandem Repeats Finder Online server (http://tandem.bu.edu/trf/trf.html) [33]. A sliding window of 200 bp was used to estimate the nucleotide diversity (Pi) of the PCGs at a step size of 20 bp by DnaSP V5 in order to evaluate the Pi value of the PCGs among seven nitidulid mitochondrial genomes [34]. The ratio of the number of nonsynonymous substitutions per nonsynonymous site (Ka) to the number of synonymous substitutions per synonymous site (Ks) of 13 PCGs for seven species of Nitidulidae was estimated using DnaSP V5 [34]. The genetic distances between seven species of Nitidulidae based on each PCG were estimated with Mega 6 [35] with the Kimura-2-parameter model. 

### 2.3. Phylogenetic Analysis

The phylogenetic analyses were performed using 13 PCGs and 2 rRNAs from 17 species of Cucujoidea (Table 1). All of the reported complete and partial mitochondrial genomes in this study were downloaded from GenBank. Standardization of data and extraction of information was conducted by PhyloSuite v1.2.1. The nucleotide sequences of the PCGs were aligned in batches with MAFFT using codon alignment and the G-INS-i (accurate) strategy. rRNAs were aligned with MAFFT version 7 online services using the Q- INS-i strategy (https://mafft.cbrc.jp/alignment/server/). Gaps and ambiguously aligned sites in the alignment were removed using Gblocks, and then by concatenating each gene into PhyloSuite. The optimal nucleotide replacement model and segmentation strategy were recommended by PartitionFinder. The best fitting models (Table S1) were selected for each partition using the “greedy” search algorithm, and were “linked” to estimated branch lengths using the Bayesian information criterion (BIC) [32].

Maximum likelihood (ML) and Bayesian inference (BI) were used for the phylogenetic analyses based on four 17-taxa datasets, namely: (1) the PCG123 matrix, including all three codon positions of protein-coding genes; (2) the PCG123R matrix, including all three codon positions of protein-coding genes and two rRNA-encoding genes; (3) the PCG12 matrix, the first and second codon positions of protein-coding genes; and (4) the PCG12R matrix, including the first and second codon positions of protein-coding genes and two rRNA-encoding genes. 

The ML phylogenetic analyses were performed using IQ-TREE V 1.6.8 [41], using an ultrafast bootstrap algorithm with 1000 replicates. The BI phylogenetic analyses were performed using MrBayes 3.2.7 [42], and 1 × 107 Markov chain Monte Carlo (MCMC) generations, sampled per 1000 generations. Convergence occurred when the average standard deviation of the split frequencies was <0.01; the first 25% of the samples were discarded as burn-in, and the remaining samples were used to generate a consensus tree and to estimate the posterior probabilities.

## 3. Results and Discussion

### 3.1. Genome Organization

The mitochondrial genomes are characterized by their asymmetric AT and GC content in the nucleotide composition. Both mitochondrial genomes show a heavy AT nucleotide bias. The AT content of the whole genome is 77.2% in *X. variegatus* (A = 39.4%, T = 37.8%, C = 13%, and G = 9.8%) and 76.4% in *Epuraea* sp. (A = 37.6%, T = 38.8%, C = 14.4%, and G = 9.3%; Table 2). Among all of the reported species of Nitidulidae, only *X. variegatus* shows a lower AT content in the AT-rich region than in the rDNAs. In addition, all of the reported Nitidulidae species show positive AT skews and negative GC skews in the whole genomes, expect for *Epuraea* sp., which has a negative AT skew (Table 3).

**Figure 1:**
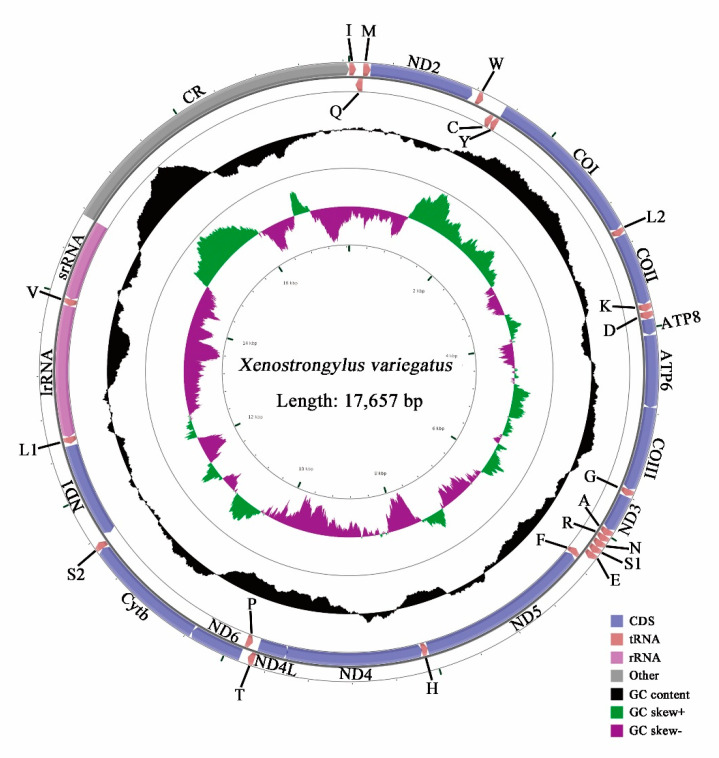
Mitochondrial map of *X. variegatus.*

**Figure 2:**
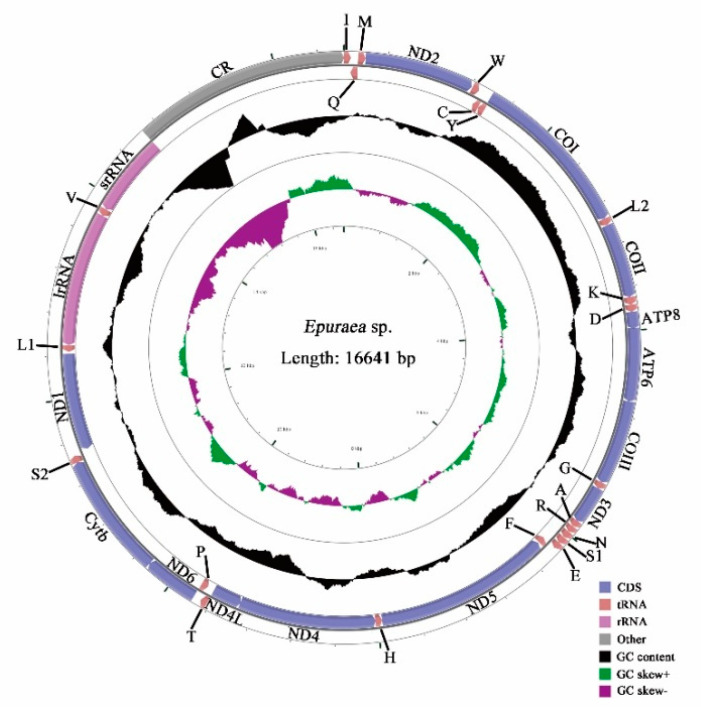
Mitochondrial map of *Epuraea* sp.

The lengths of the complete mitochondrial genome are 17,657 bp in *X. variegatus* and 16,641 bp in *Epuraea* sp., the length of the former is longer than that reported for Nitidulidae (Table 3) because of the differences in the number of AT-repeats in the AT-rich region. The mitochondrial genomes of both species consist of closed, circular, double-stranded DNA molecules (Figure 1 and Figure 2), and contain 37 genes, including 13 PCGs, 22 tRNAs, 2 rDNAs, and a AT-rich region. While four PCGs (*nad1*, *nad4*, *nad4L,* and *nad5*), eight tRNAs (*Q*, *C*, *Y*, *F*, *H*, *P*, *L1.* and *V*), and two rRNAs (*lrRNA* and *srRNA*) are encoded in the heavy strand, the others are encoded in the light strand (Table 4). The sequence of genes is consistent with the reference mitochondrial genome arrangement and with other Nitidulidae.

Apart from the AT-rich region, there are 197 bp spacers across nine gene intervals ranging from 1–114 bp in *X. variegatus,* and 62 bp spacers across eight gene intervals ranging from 1–19 bp in *Epuraea* sp. The longest intergenic spacer is located between *trnW* and *trnC* in *X. variegatus*, and *nad1* and *trnL1* in *Epuraea* sp, while in *A. tumida* the longest is 18 bp between *trnL2* and *cox2*. In *C. dimidiatus*, *C. pilosellus,* and *E. guttata*, there are 24 bp, 107 bp, and 79 bp intergenic spacers between *trnW* and *trnC*, respectively. Gene overlaps are found at the junctions of 11 pairs of genes ranging from 1–10 bp in *X. variegatus* and 1–9 bp in *Epuraea* sp., with the longest overlap located between *nad4* and *trnT* in *X. variegatus*, *trnY,* and *cox1* in *Epuraea* sp, *A. tumida, C. dimidiatus*, *C. pilosellus,* and *E. guttata*. 

### 3.2. Protein-coding Genes (PCGs)

The total length of all 13 PCGs of *X. variegatus* is 11,046 bp and of *Epuraea* sp. is 11,097 bp, accounting for 62.56% and 66.68% of the total length of their mitochondrial genomes, respectively (Table 2). The start and stop codons were determined based on the reference sequences. Most PCGs start with a typical start codon ATN (ATC, ATG, ATA, and ATT), except for *nad1*, which starts with the unusual start codon TTG in *A. tumida*, *E. guttata,* and an unidentified Nitidulidae sp. Correspondingly, the PCGs ended with the stop codons TAA and TAG, whereas an incomplete stop codon, T, was found in *cox1*, *cox2*, *cox3*, *atp8*, *nad4*, and *nad5* in Nitidulidae (Table 5). Such incomplete stop codons are common in insects and may result from post-transcriptional polyadenylation [43]. Furthermore, the stop codon TAA is used more frequently than TAG, and all seven Nitidulidae have *cox1*, at least, ending in an incomplete stop codon T.

**Figure 3:**
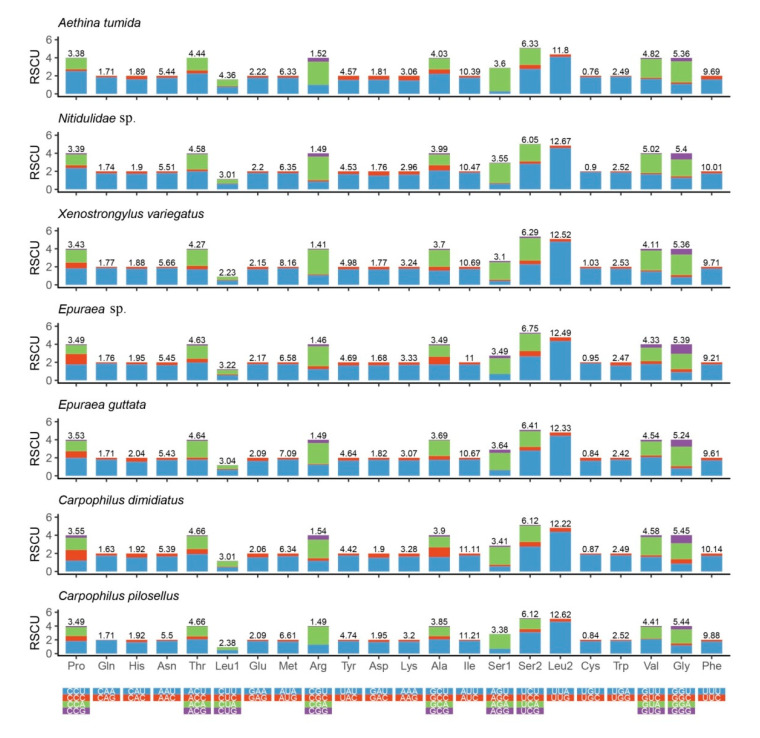
Relative synonymous codon usage (RSCU) of the mitochondrial DNA protein-coding genes (PCGs) of seven nitidulid species.

The total AT ratios of 13 PCGs are 77.0% in *X. variegatus* (A = 34.0%, T = 43.0%, C = 13.0%, and G = 9.8%) and 74.9% in *Epuraea* sp. (A = 32.0%, T = 42.9%, C = 12.9%, and G = 12.2%). Both species show negative AT skews (−0.116 in *X. variegatus* and −0.146 in *Epuraea* sp.). *X. variegatus* shows no CG skew (0) and *Epuraea* sp. shows a negative CG skew (−0.026) (Table 2). The first codon position AT content (72.3% in *X. variegatus* and 71.1% in *Epuraea* sp.) is higher than that of the second codon position (68.9% in *X. variegatus* and 67.9% *Epuraea* sp.) and is much lower than that of the third codon position (89.8% in *X. variegatus* and 85.5% in *Epuraea* sp.). The relative synonymous codon usage (RSCU) is shown in Figure 3. UUA (*Leu*), AUU (*Ile*), UUU (*Phe*), UCU (*Ser 2*), and AUA (*Met*) are the most frequently used codons in both species, which is highly consistent with the previously reported frequencies in Nitidulidae. As indicated by these results, nearly all of them consist of A and U, and contribute to the high AT content of PCGs.

### 3.3. Transfer and Ribosomal RNAs

The total length of all 22 tRNAs of *X. variegatus* is 1454 bp and of *Epuraea* sp. is 1445 bp, which is within the previously reported range for Nitidulidae, accounting for 8.23% and 8.68% of the total length of their mitochondrial genomes, respectively. The total AT percent is 78.2% (A = 39.6%, T = 38.6%, C = 9%, and G = 12.8%) for *X. variegatus* and 75.7% (A = 39.4%, T = 36.3%, C = 10.9%, and G = 13.4%) for *Epuraea* sp. Both species show positive AT skews (0.013 in *X. variegatus* and 0.041 in *Epuraea* sp.) and CG skews (0.174 in *X. variegatus* and 0.103 in *Epuraea* sp.) (Table 2). The length of each tRNA is between 63 bp (*trnY* and *trnR*) and 71 bp (*trnK*) in *X. variegatus* and between 62 bp (*trnC* and *trnR*) and 70 bp (*trnK*) in *Epuraea* sp. (Table 4).

**Figure 4:**
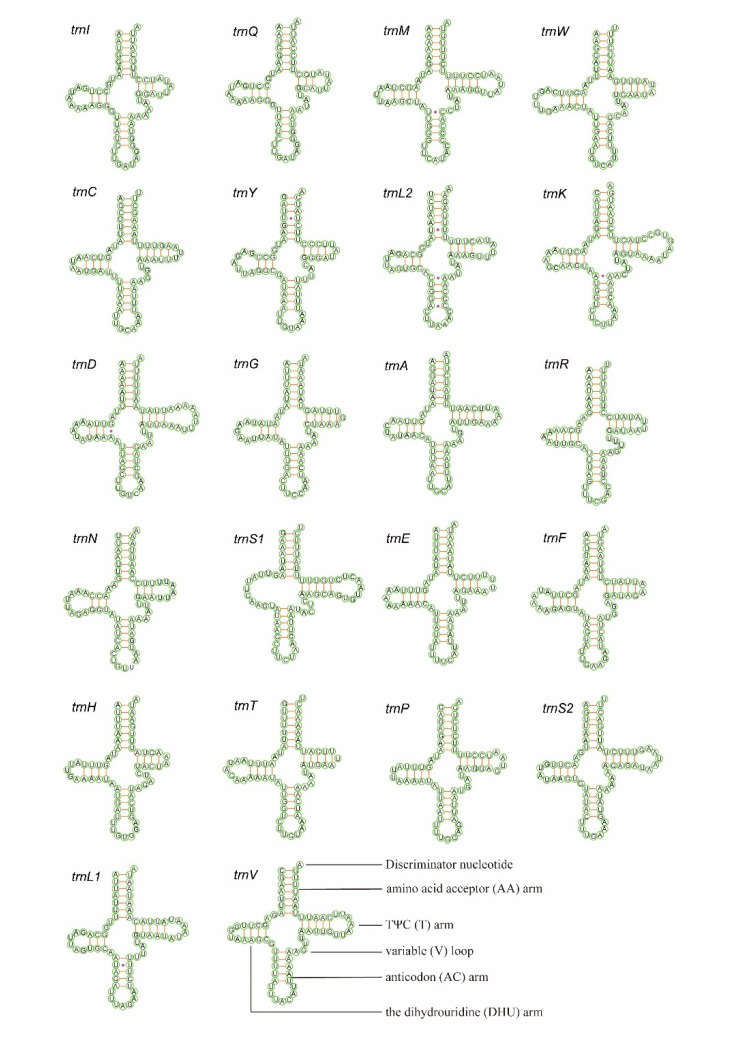
Inferred secondary structure for the tRNAs of *X. variegatus*.

**Figure 5:**
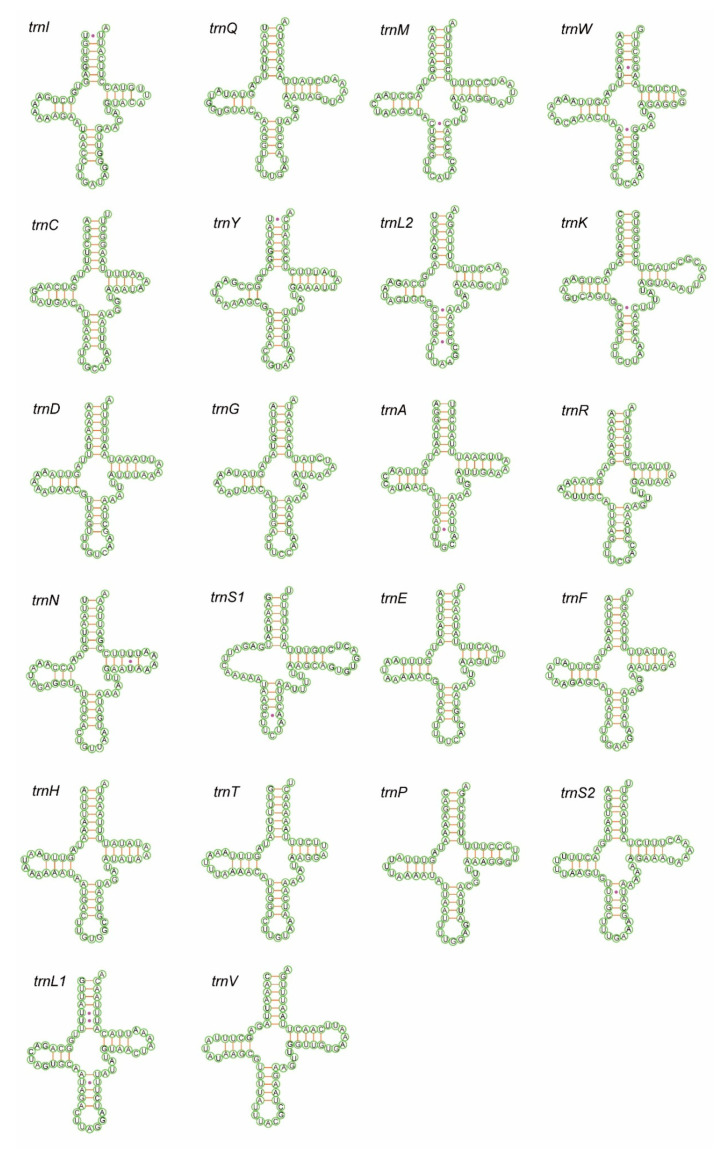
Inferred secondary structure for the tRNAs of *Epuraea* sp.

Nearly all tRNAs can be folded into the typical clover-leaf structure, except for *trnS1,* which in both shows a reduced dihydrouridine (DHU) arm. The size of the anticodon (AC) arm and the amino acid acceptor (AA) arm are consistently 5 bp and 7 bp, respectively. The TΨC arm and DHU arm are variable: *trnW*, *trnF, trnH,* and *trnT* in both species; *trnG* in *X. variegatus;* and *trnR* in *Epuraea* sp. all lack the TΨC-loop. The *trnS1* in both species lack the dihydorouridine (DHU) arm, which has been reported in other metazoans [44,45,46,47,48,49]. The length of the AC-loop is normally seven nucleotides, except for *trnA* in *X. variegatus,* which is six nucleotides. The *trnS1* and *trnA* in *Epuraea* sp. have five nucleotides and the DHU loop ranges from 2–4 bp. The TΨC loop ranges from 3–5 bp in both species. The DHU-loop ranges from 3–9 nucleotides in *Epuraea* sp. and 3–8 nucleotides in *X. variegatus*. There are a total of 27 mismatched base pairs in *X. variegatus* of six types (U-U, U-G, A-G, A-C, U-C, and A-A) and 33 mismatched base pairs of six types (U-U, U-G, C-C, A-G, A-C, and U-C) found in *Epuraea* sp (Figure 4 and Figure 5).

The *rRNAL* and *rRNAS* are located between *trnL1* and *trnV*, and *trnV* and the AT-rich region with lengths in *X. variegatus* of 1291 bp and 788 bp, but 1300 bp and 781 bp in *Epuraea* sp. The total rRNAs show a negative AT skew (−0.053 in *X. variegatus* and −0.063 in *Epuraea* sp.) and a positive CG skew (0.296 in *X. variegatus* and 0.353 in *Epuraea* sp.). The AT content in *X. variegatus* is 81.3% and 78.8% in *Epuraea* sp (Table 3). Therefore, rRNAs are highly conserved in the Nitidulidae for length, AT content, and location.

### 3.4. AT-rich Region

**Figure 6:**
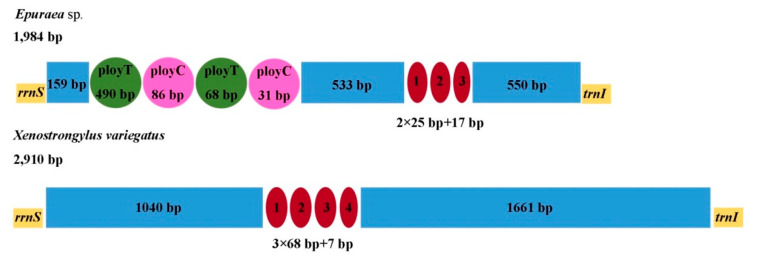
Structures of AT-rich region in mitogenomes of *Epuraea* sp. and *X. variegatus*. The dark red ellipses are the tandem repeat regions, the blue blocks indicate non-repeat regions, the green circles are the poly-T stretches, and the purple circles are poly-C stretches.

The assumed control region (the AT-rich region) is the major noncoding region in the mitochondrial genome. It is located between *rrnS* and *trnI*, and plays a regulatory role in the transcription and replication of the mtDNA [50,51,52,53,54]. The lengths of the AT-rich region of *X. variegatus* and *Epuraea* sp. are 2910 bp and 1984 bp, respectively (Figure 6). Both are longer than those previously reported for Nitidulidae. The AT contents of these regions are 74.6% and 82.6% in *X. variegatus* and *Epuraea* sp., respectively. The AT-rich regions in both species show negative AT skews (−0.078 in *X. variegatus* and −0.302 in *Epuraea* sp.) and negative CG skews (−0.064 in *X. variegatus* and −0.397 in *Epuraea* sp.). Both species have different lengths of tandem repeat, located at positions 1041 bp to 1660 bp in *X. variegatus* and 1368 bp to 1436 bp in *Epuraea* sp., respectively. Moreover, two poly-T stretches and two poly-C stretches are found near *rrnS* in *Epuraea* sp., which may be the origin of the DNA replication minor strand [51].

### 3.5. Nucleotide Analyses

**Figure 7:**
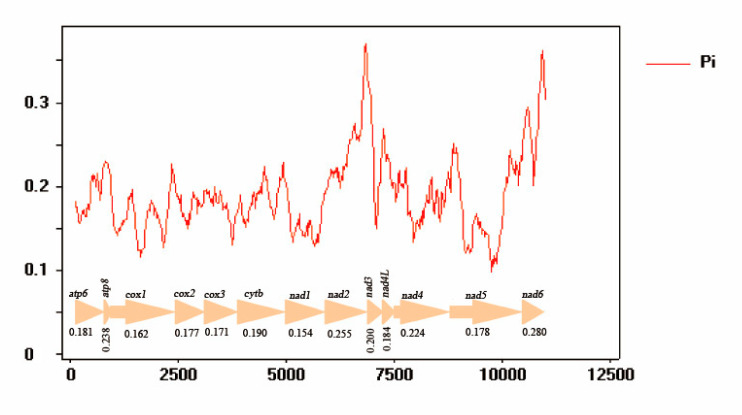
Sliding window analyses of 13 PCGs among seven nitidulid mitogenomes. The red line shows the value of nucleotide diversity (Pi) in a sliding window analysis (a sliding window of 200 bp with the step size of 20 bp); the Pi value of each gene is shown under the gene name.

The nucleotide diversity calculated for 13 PCGs of the seven Nitidulidae are shown in Figure 7. The results indicate that different genes have different nucleotide diversity values. In all PCGs, *nad6* (Pi = 0.280) shows the highest nucleotide diversity values, next to *nad2* (Pi = 0.255) and *atp8* (Pi = 0.238). However, *cox1* (Pi = 0.162) and *nad1* (Pi = 0.154) show lower nucleotide diversity values and are the most conserved of the mitochondrial PCGs (Figure 7). 

**Figure 8:**
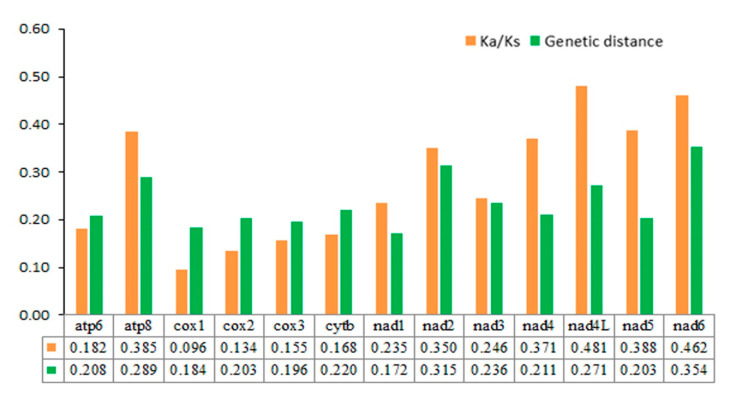
Genetic distance and non-synonymous (Ka) to synonymous (Ks) substitution rates of 13 PCGs among seven nitidulid species.

Pairwise comparisons of the genetic distances show consistent results: *nad6* (0.354) and *nad2* (0.315) have greater distances and a faster evolution, while *nad1* (0.172) and *cox1* (0.184) represent shorter distances and a slower evolution. The average nonsynonymous (Ka) and synonymous (Ks) replacement rates of the 13 PCGs in seven mitochondrial genomes are estimated to be in the range of 0.096–0.481, indicating that all PCGs are under purifying selection. In addition, *cox1* (0.096) exhibits the strongest purifying selection and shows the lowest evolutionary rate. In contrast, the substitution rates of *nad4L* (0.481) and *nad6* (0.462) are much higher than in other PCGs, suggesting that they may be under a relaxed purifying selection (Figure 8). This suggests that the latter gene may be most suitable for resolving phylogenetic relationships among closely related species.

### 3.6. Phylogenetic Analysis

**Figure 9:**
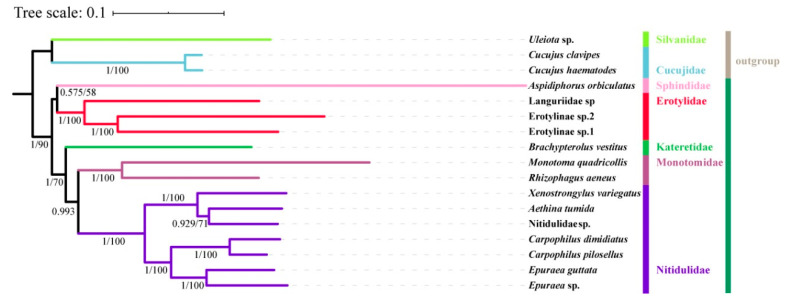
Phylogenetic tree produced from Maximum likelihood (ML) and Bayesian inference (BI) analyses based on PCG12R. The numbers on branches are bootstrap value (BS) and Bayesian posterior probabilities (PP).

The phylogenetic analyses in this study were based on four datasets (PCG123, PCG123R, PCG12, and PCG12R) including 17 species of Cucujoidea. The partitioning schemes and models for the four datasets are listed in Tables S1 and S2. Eight tree topologies were constructed according to the ML and BI analysis (Figure 9 and Figures S1–S6). Although the tree topologies were not completely consistent among the analyses, all of the results support the monophyly of Nitidulidae and a sister-group relationship of Kateretidae + (Monotomidae + Nitidulidae).

Both BI and ML methods based on four different datasets strongly support the monophyly of Nitidulidae (Nitidulinae + (Carpophilinae + Epuraeinae)), which is consistent with previous studies of Cline and Lee [1,21,25]. In the present study, Kateretidae consistently forms a sister-group with Monotomidae + Nitidulidae, forming a monophyletic clade with moderate support (bootstrap value (BS) = 70 and Bayesian posterior probabilities (PP) = 1). The sister relationship of Nitidulidae to Monotomidae is supported by high posterior probabilities in BI trees (PP = 0.993). This result is consistent with that of Hunt [28], but contradicts most previous phylogenetic analyses based on morphological characters [25,26,27] and gene fragments [1,2,21], which all support the Nitidulidae sister to Kateretidae. Considering that only a few taxa are included in this study, more species need to be sequenced and the mitochondrial data need to be combined with data from nuclear genes and morphology in order to provide a more robust phylogeny of Nitidulidae and the related families.

## 4. Conclusions

New complete mitochondrial genomes of two nitidulid species, *X. variegatus* and *Epuraea* sp., are provided. Comparative analyses of the available Nitidulidae mitochondrial genomes show that they are highly conserved in terms of their genome size, base content and composition, codon usage, and secondary structures of tRNAs. The results of the phylogenetic analyses confirm the monophyly of Nitidulidae and support the sister relationship of Kateretidae + (Monotomidae + Nitidulidae). This indicates that mitochondrial data can help resolve phylogenetic relationships at different levels in the taxonomic hierarchy. Although some differences between the present results and previously published phylogenies of this group of beetles may be due to differences in the taxon sampling and phylogenetic analysis methods, the present study indicates that mitochondrial genome sequencing can contribute to an improved understanding of the phylogenetic relationships among and within the Cucujoidea.

## Figures and Tables

**Table 1:** Summary of the mitogenomic sequence information used in the present study.

Family	Species	Accession Number	Reference
Sphindidae	*Aspidiphorus orbiculatus*	KT780625	Unpublished
Erotylidae	Languriidae sp.	MG193464	[36]
	Erotylinae sp1	MH836601	[37]
	Erotylinae sp2	MH789736	[37]
Monotomidae	*Monotoma quadricollis*	KX035132	Unpublished
	*Rhizophagus aeneus*	KX087340	Unpublished
Kateretidae	*Brachypterolus vestitus*	KX087245	Unpublished
Nitidulidae	*Nitidulidae* sp	MH789742	[37]
	*Aethina tumida*	NC_036104	[38]
	*Xenostrongylus* *variegatus*	MW044620	This study
	*Epuraea guttata*	KX087289	Unpublished
	*Carpophilus dimidiatus*	NC_046036	[39]
	*Carpophilus pilosellus*	MN604383	[39]
	*Epuraea* sp.	MW044619	This study
Silvanidae	*Uleiota* sp.	KX035149	Unpublished
Cucujidae	*Cucujus clavipes*	GU176341	[40]
	*Cucujus haematodes*	KX087268	Unpublished

**Table 2:** Nucleotide composition of mitogenomes of *X. variegatus* and *Epuraea* sp.

Regions	Size (bp)	T(U)	C	A	G	AT(%)	GC(%)	AT Skew	GC Skew
*X*. *variegatus*									
Full genome	17,657	37.8	13	39.4	9.8	77.2	22.8	0.021	−0.141
PCGs	11,046	43	11.5	34	11.5	77	23	−0.116	0
1st codon position	3682	37.2	10.7	35.1	17.1	72.3	27.8	−0.029	0.229
2nd codon position	3682	47.3	17.7	21.6	13.4	68.9	31.1	−0.374	−0.136
3rd codon position	3682	44.4	6.2	45.4	4	89.8	10.2	0.012	−0.207
tRNAs	1454	38.6	9	39.6	12.8	78.2	21.8	0.013	0.174
rRNAs	2079	42.8	6.6	38.5	12.1	81.3	18.7	−0.053	0.296
AT-rich region	2910	40.2	13.5	34.4	11.9	74.6	25.4	−0.078	−0.064
*Epuraea* sp.									
Full genome	16,641	38.8	14.4	37.6	9.3	76.4	23.7	−0.015	−0.216
PCGs	11,097	42.9	12.9	32	12.2	74.9	25.1	−0.146	−0.026
1st codon position	3699	36.7	11.9	34.4	17	71.1	28.9	−0.032	0.179
2nd codon position	3699	46.7	18.3	21.2	13.7	67.9	32	−0.376	−0.143
3rd codon position	3699	45.2	8.5	40.3	6	85.5	14.5	−0.057	−0.175
tRNAs	1445	36.3	10.9	39.4	13.4	75.7	24.3	0.041	0.103
rRNAs	2081	41.9	6.9	36.9	14.4	78.8	21.3	−0.063	0.353
AT-rich region	1984	53.8	12.1	28.8	5.2	82.6	17.3	−0.302	−0.397

**Table 3:** Nucleotide composition of the Nitidulidae mitochondrial genomes: *E. guttata* (*E1*), *Epuraea* sp. (*E2*), *C. dimidiatus* (*C1*), *C. pilosellus* (*C2*), Nitidulidae sp. (N), *A. tumida* (*A*), and *X. variegatus* (*X*).

Species	Whole Genome		AT Skew	GC Skew	PCGs		tRNAs		rRNAs		A + T-Rich Region	
	Size (bp)	AT (%)			Size (bp)	AT (%)	Size (bp)	AT (%)	Size (bp)	AT (%)	Size (bp)	AT (%)
*E1*	16,021	76.5	0.043	−0.19	11,073	75.7	1451	75.7	2081	76.4	-	-
*E2*	16,641	76.4	−0.015	−0.216	11,097	74.9	1445	75.8	2081	78.8	1984	82.6
*C1*	15,717	75.2	0.038	−0.202	11,094	74.5	1441	74.9	2061	75	1057	83.6
*C2*	15,686	77.2	0.027	−0.177	11,103	76.5	1442	76.5	2079	77.5	944	86.7
*N*	17,432	78.4	0.036	−0.183	11,091	76.3	1443	78.2	2073	80.3	-	-
*A*	16,576	76.9	0.034	−0.223	11,109	75.4	1460	77.2	2064	79.5	-	-
*X*	17,657	77.2	0.021	−0.141	11,046	77	1454	78.2	2079	81.3	2910	74.6

**Table 4:** Mitogenomic organization of *X. variegatus* and *Epuraea* sp.

	Position		Size (bp)	Intergenic Nucleotides	Codon		Strand
	From	To			Start	Stop	
*X*. *variegatus/E*. sp.							
*trnI*	1/1	64/63	64/63				+/+
*trnQ*	62/61	130/129	69/69	−3/−3			−/−
*trnM*	131/129	199/197	69/69	/−1			+/+
*nad2*	200/198	1174/1205	975/1008		ATT/ATT	TAA/TAA	+/+
*trnW*	1202/1214	1268/1280	67/67	27/8			+/+
*trnC*	1383/1284	1446/1345	64/62	114/3			−/−
*trnY*	1448/1346	1510/1410	63/65	1/			−/−
*cox1*	1503/1403	3042/2942	1540/1540	−8/−8	ATT/ATC	T/T	+/+
*trnL2*	3043/2943	3107/3007	65/65				+/+
*cox2*	3108/3008	3780/3695	673/688		ATT/ATT	T/T	+/+
*trnK*	3781/3696	3851/3765	71/70				+/+
*trnD*	3855/3766	3924/3831	70/66	3/			+/+
*atp8*	3925/3832	4069/3987	145/156		ATC/ATC	T/TAG	+/+
*atp6*	4076/3981	4747/4655	672/675	6/−7	ATA/ATG	TAA/TAA	+/+
*cox3*	4747/4655	5533/5438	787/784	−1/−1	ATG/ATG	T/TAG	+/+
*trnG*	5534/5439	5597/5501	64/63				+/+
*nad3*	5604/5502	5951/5855	348/354	6/	ATT/ATT	TAG/T	+/+
*trnA*	5950/5854	6015/5917	66/64	−2/−2			+/+
*trnR*	6015/5918	6077/5979	63/62	−1/			+/+
*trnN*	6077/5980	6142/6046	66/67	−1/			+/+
*trnS1*	6143/6047	6209/6113	67/67				+/+
*trnE*	6210/6114	6273/6176	64/63				+/+
*trnF*	6272/6175	6336/6239	65/65	−2/−2			−/−
*nad5*	6337/6249	8053/7953	1717/1705	/9	ATA/ATT	T/TAG	−/−
*trnH*	8051/7954	8114/8018	64/65	−3/			−/−
*nad4*	8112/8016	9444/9342	1333/1327	−3/−3	ATT/ATA	T/T	−/−
*nad4L*	9435/9339	9722/9623	288/285	−10/−4	ATG/ATG	TAA/TAA	−/−
*trnT*	9725/9626	9789/9689	65/64	2/2			+/+
*trnP*	9790/9690	9854/9755	65/66				−/−
*nad6*	9859/9760	10,359/10,263	501/504	4/4	ATA/ATA	TAA/TAA	+/+
*cytb*	10,359/10,263	11,498/11,405	1140/1143	−1/−1	ATG/ATG	TAG/TAG	+/+
*trnS2*	11,497/11,404	11,564/11,471	68/68	−2/−2			+/+
*nad1*	11,582/11,489	12,514/12,421	933/933	17/17	ATT/ATT	TAG/TAG	−/−
*trnL1*	12,534/12,441	12,600/12,505	67/65	19/19			−/−
*rrnL*	12,601/12,506	13,891/13,805	1291/1300				−/−
*trnV*	13,892/13,806	13,959/13,875	68/70				−/−
*rrnS*	13,960/13,877	14,747/14,657	788/781	/1			−/−
AT-rich region	14,748/14,658	17,657/16,641	2910/1984				+/+

**Table 5:** Start and stop codons of the mitochondrial genomes: *E. guttata* (*E1*), *Epuraea* sp. (*E2*), *C. dimidiatus* (*C1*), *C. pilosellus* (*C2*), Nitidulidae sp. (*N*), *A. tumida* (*A*), and *X. variegatus* (*X*).

Gene	Start Codon/Stop Codon						
	*E1*	*E2*	*C1*	*C2*	*N*	*A*	*X*
*nad2*	ATT/TAA	ATT/TAA	ATT/TAA	ATT/TAA	ATT/T	ATT/TAA	ATT/TAA
*cox1*	ATT/T	ATC/T	ATT/T	ATT/T	ATT/T	ATA/T	ATT/T
*cox2*	ATA/T	ATT/T	ATC/T	ATT/T	ATT/TAG	ATT/T	ATT/T
*atp8*	ATT/TAG	ATC/TAG	ATC/TAG	ATC/TAG	ATG/TAA	ATT/TAG	ATC/T
*atp6*	ATG/TAA	ATG/TAA	ATG/TAA	ATA/TAA	ATG/TAA	ATA/TAA	ATA/TAA
*cox3*	ATG/T	ATG/T	ATG/T	ATG/T	ATT/TAA	ATG/T	ATG/T
*nad3*	ATA/TAG	ATT/TAG	ATT/TAG	ATT/TAG	ATT/TAA	ATA/TAG	ATT/TAG
*nad5*	ATA/T	ATT/T	ATT/T	ATT/T	TAG/TAA	ATA/T	ATA/T
*nad4*	ATG/TAA	ATA/T	ATG/T	ATG/T	ATG/TAA	ATG/T	ATT/T
*nad4L*	ATG/TAA	ATG/TAA	ATG/TAA	ATG/TAA	ATT/TAA	ATG/TAA	ATG/TAA
*nad6*	ATC/TAA	ATA/TAA	ATA/TAA	ATA/TAA	ATG/TAG	ATA/TAA	ATA/TAA
*Cytb*	ATA/TAG	ATG/TAG	ATG/TAG	ATG/TAG	TTG/TAG	ATG/TAA	ATG/TAG
*nad1*	AAC/ATC	ATT/TAG	ATA/TAG	ATG/TAG	ATT/TAA	TTG/TAG	ATT/TAG

## Supplementary Materials

The following are available online at https://www.mdpi.com/2075-4450/11/11/779/s1, Figure S1: Phylogenetic tree produced from the BI method based on a PCG12 dataset. Figure S2: Phylogenetic tree produced from the ML method based on a PCG12 dataset. Figure S3: Phylogenetic tree produced from the BI method based on a PCG123 dataset. Figure S4: Phylogenetic tree produced from the ML method based on a PCG123 dataset. Figure S5: Phylogenetic tree produced from the BI method based on a PCG123R dataset. Figure S6: Phylogenetic tree produced from the ML method based on a PCG123R dataset. Table S1: Best partitioning scheme and nucleotide substitution models for different datasets selected by PartitionFinder.
