# Supplementary material for: Characterization of the Complete Mitochondrial Genomes from Two Nitidulid Pests with Phylogenetic Implications
Source: Insects. 2020 Nov 11;11(11):779. doi: 10.3390/insects11110779 (PMC7697951; doi:10.3390/insects11110779)
Supplement: Supplementary file 1 [file insects-11-00779-s001.pdf]

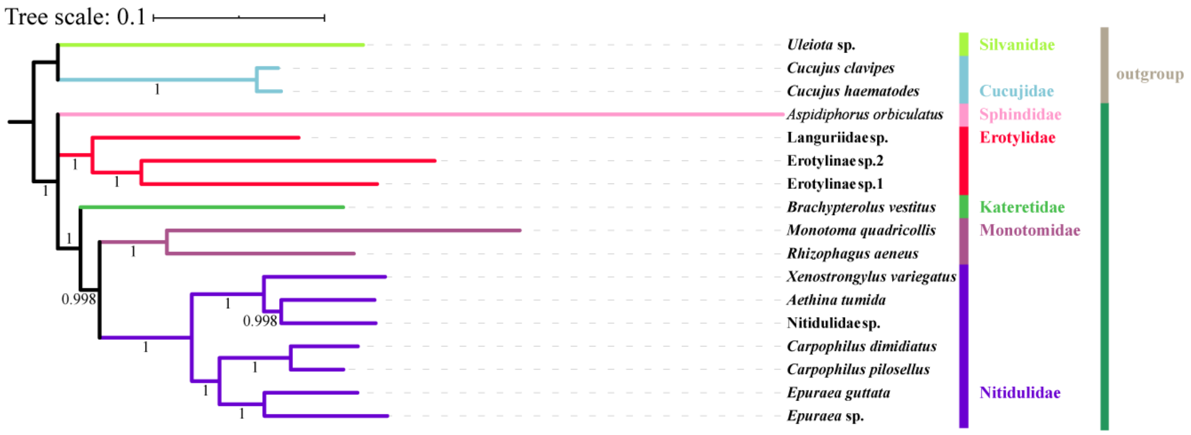

Figure S1. Phylogenetic tree produced from the BI method based on a PCG12 dataset.

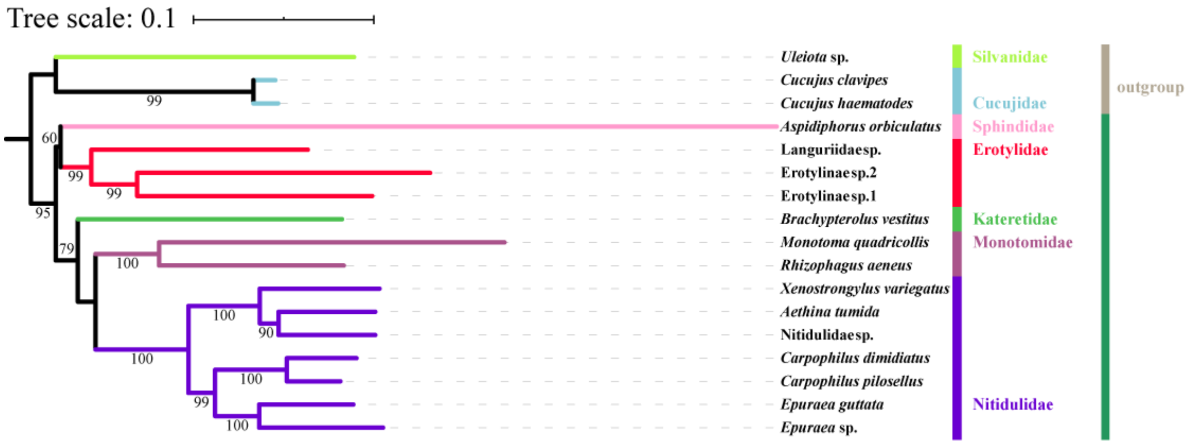

Figure S2. Phylogenetic tree produced from the ML method based on a PCG12 dataset.

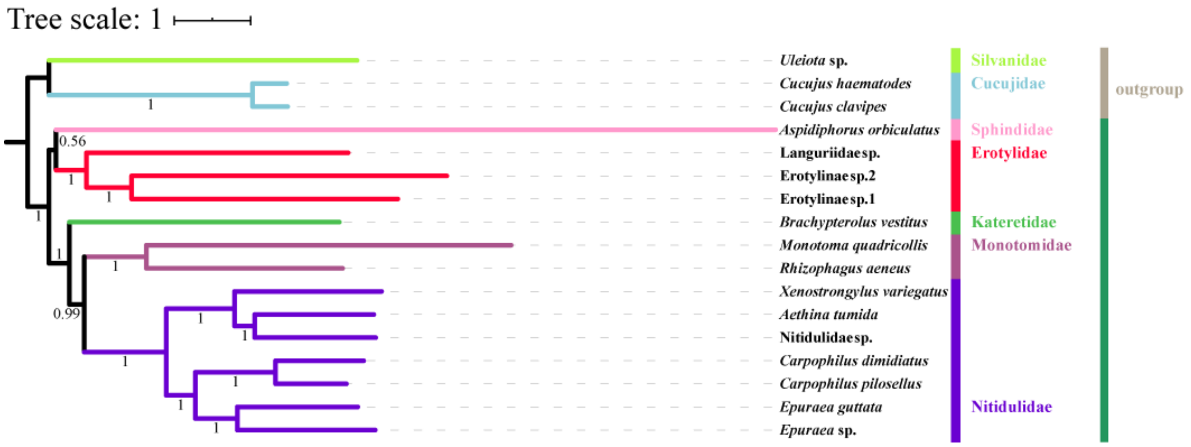

Figure S3. Phylogenetic tree produced from the BI method based on a PCG123 dataset.

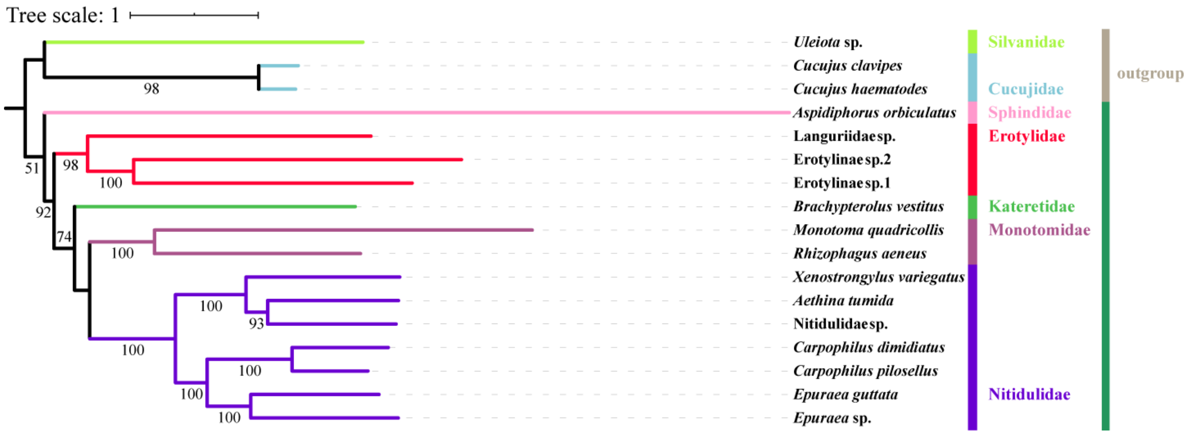

Figure S4. Phylogenetic tree produced from the ML method based on a PCG123 dataset.

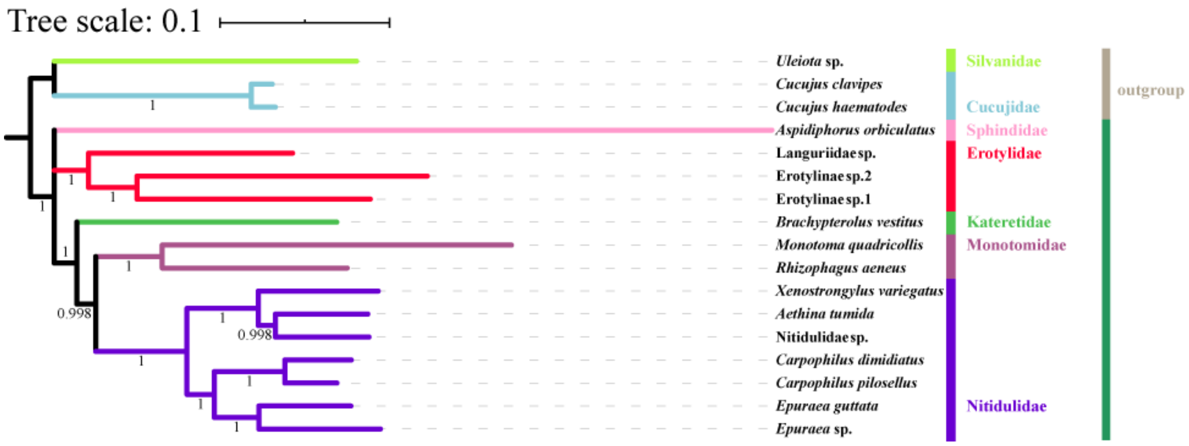

Figure S5. Phylogenetic tree produced from the BI method based on a PCG123R dataset.

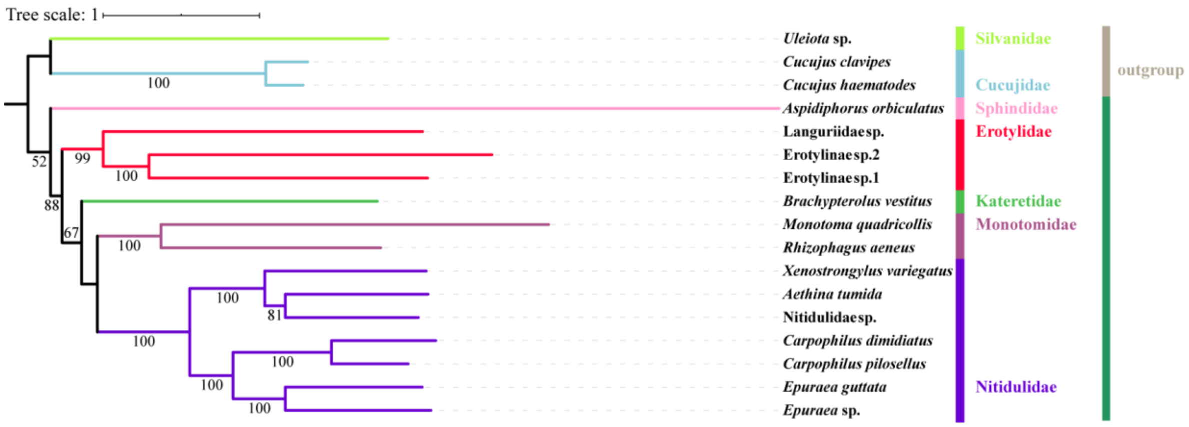

Figure S6. Phylogenetic tree produced from the ML method based on a PCG123R dataset.

Table S1. Best partitioning scheme and nucleotide substitution models for different datasets selected by PartitionFinder.

	Dataset	Optional Partition	Best Model	Subset Partitions
**BI**	PCG123	Patition1	GTR+I+G	*atp6_pos1, cox3_pos1, cytb_pos1, cox2_pos1, cox1_pos1*
		Patition2	GTR+I+G	*atp6_pos2, cox1_pos2, cox2_pos2, cox3_pos2, cytb_pos2*
		Patition3	GTR+G	*nad6_pos3, atp6_pos3, nad3_pos3, cox3_pos3, cytb_pos3, atp8_pos3, cox2_pos3*
		Patition4	GTR+I+G	*nad3_pos1, nad2_pos1, nad6_pos1, atp_pos1*
		Patition5	GTR+G	*nad6_pos2, nad3_pos2, nad2_pos2, atp8_pos2*
		Patition6	HKY+I+G	*cox1*
		Patition7	GTR+I+G	*nad1_pos1, nad5_pos1, nad4_pos1, nad4L_pos1*
		Patition8	GTR+I+G	*nad1_pos2, nad4_pos2, nad5_pos2, nad4L_pos2*
		Patition9	GTR+G	*nad1_pos3, nad4L_pos3, nad5_pos3, nad4_pos3*
		Patition10	HKY+G	*nad2_pos3*
	PCG12	Patition1	GTR+I+G	*atp6, cox3, cytb*
		Patition2	GTR+I+G	*nad6, atp8, nad3, nad2*
		Patition3	GTR+I+G	*cox1, cox2*
		Patition4	GTR+I+G	*nad1, nad4L, nad5, nad4*
	PCG123R	Patition1	GTR+I+G	*atp6_pos1, cox3_pos1, cytb_pos1, cox2_pos1, cox1_pos1*
		Patition2	GTR+I+G	*atp6_pos2, cox1_pos2, cox2_pos2, cox3_pos2, cytb_pos2*
		Patition3	GTR+G	*nad6_pos3, atp6_pos3, nad3_pos3, atp8_pos3, cox2_pos3, cox3_pos3, cytb_pos3*
		Patition4	GTR+I+G	*nad3_pos1, nad2_pos1, nad6_pos1, atp8_pos1*
		Patition5	GTR+G	*nad3_pos2, nad2_pos2, nad6_pos2, atp8_pos2*
		Patition6	HKY+I+G	*cox1_pos3*
		Patition7	GTR+I+G	*nad1_pos1, rrnaS, mad5_pos1, nad4_pos1, nad4L_pos1*
		Patition8	GTR+I+G	*nad1_pos2, nad4_pos2, nad5_pos2, nad4L_pos2*
		Patition9	GTR+G	*nad1_pos3, nad4_pos3, nad5_pos3, nad4L_pos3*
		Patition10	HKY+G	*nad2_pos3*
		Patition11	GTR+I+G	*rrnaL*
	PCG12R	Patition1	GTR+I+G	*atp6, cox3, cytb, cox1, cox2*
		Patition2	GTR+I+G	*nad6, atp8, nad3, nad2*
		Patition3	GTR+I+G	*nad1, nad4L, nad5, nad4*
		Patition4	GTR+I+G	*rrnaL*

		Patition5	GTR+I+G	*rrnaS*

	Dataset	Optional Partition	Subset Partitions	Best Model
**ML**	PCG123	Patition1	GTR+I+G	*atp6_pos1, cox3_pos1, cytb_pos1*
		Patition2	TVM+G	*atp6_pos2*
		Patition3	TRN+G	*atp6_pos3, nad3_pos3*
		Patition4	GTR+I+G	*nad2_pos1, atp8_pos1*
		Patition5	TVM+G	*nad6_pos2, atp8_pos2*
		Patition6	TRN+I+G	*cytb_pos3, cox3_pos3, atp8_pos3, cox2_pos3*
		Patition7	GTR+G	*cox2_pos1, cox1_pos1*
		Patition8	GTR+I+G	*cox1_pos2*
		Patition9	HKY+I+G	*cox1_pos3*
		Patition10	GTR+I+G	*cytb_pos2, cox3_pos2, cox2_pos2*
		Patition11	TVM+I+G	*nad1_pos1*
		Patition12	GTR+I+G	*nad1_pos2*
		Patition13	HKY+G	*nad1_pos3*
		Patition14	TVM+I+G	*nad3_pos2, nad2_pos2*
		Patition15	GTR+G	*nad2_pos3*
		Patition16	GTR+I+G	*nad3_pos1*
		Patition17	TVM+I+G	*nad5_pos1, nad4_pos1, nad4L_pos1*
		Patition18	GTR+I+G	*nad5_pos2, nad4_pos2, nad4L_pos2*
		Patition19	GTR+G	*nad4L_pos3, nad4_pos3, nad5_pos3*
		Patition20	GTR+G	*nad6_pos1*
		Patition21	HKY+G	*nad6_pos3*
	PCG12	Patition1	GTR+I+G	*atp6, cox3, cytb*
		Patition2	TVM+I+G	*atp8, nad2*
		Patition3	GTR+I+G	*cox1*
		Patition4	GTR+G	*cox2*
		Patition5	GTR+I+G	*nad1*
		Patition6	TIM+G	*nad3*
		Patition7	GTR+I+G	*nad4, nad5*
		Patition8	TIM+I+G	*nad4L*

	Patition9	TVM+G	*nad6*
	Patition1	GTR+I+G	*atp6_pos1, cox3_pos1, cytb_pos1*
	Patition2	TVM+G	*atp6_pos2*
	Patition3	TRN+G	*atp6_pos3, nad3_pos3*
	Patition4	GTR+I+G	*nad2_pos1, atp8_pos1*
	Patition5	TVM+G	*nad6_pos2, atp8_pos2*
	Patition6	TRN+I+G	*cytb_pos3, cox3_pos3, atp8_pos3, cox2_pos3*
	Patition7	GTR+G	*cox2_pos1, cox1_pos1*
	Patition8	GTR+I+G	*cox1_pos2*
	Patition9	HKY+I+G	*cox1_pos3*
	Patition10	GTR+I+G	*cytb_pos2, cox3_pos2, cox2_pos2*
	Patition11	TVM+I+G	*nad1_pos1*
PCG123R	Patition12	GTR+I+G	*nad1_pos2*
	Patition13	HKY+G	*nad1_pos3*
	Patition14	TVM+I+G	*nad3_pos2, nad2_pos2*
	Patition15	GTR+G	*nad2_pos3*
	Patition16	GTR+I+G	*nad3_pos1*
	Patition17	TVM+I+G	*nad5_pos1, nad4_pos1, nad4L_pos1*
	Patition18	GTR+I+G	*nad5_pos2, nad4_pos2, nad4L_pos2*
	Patition19	GTR+G	*nad4L_pos3, nad4_pos3, nad5_pos3*
	Patition20	GTR+G	*nad6_pos1*
	Patition21	HKY+G	*nad6_pos3*
	Patition22	GTR+I+G	*rrnaS*
	Patition23	GTR+I+G	*rrnaL*
	Patition1	GTR+I+G	*atp6, cox3, cytb*
	Patition2	GTR+I+G	*atp8, nad2*
	Patition3	GTR+I+G	*cox1*
PCG12R	Patition4	GTR+G	*cox2*
	Patition5	GTR+I+G	*nad1*
	Patition6	TIM+G	*nad3*
	Patition7	GTR+I+G	*nad4, nad5*

Patition8	TIM+I+G	*nad4L*
Patition9	TVM+G	*nad6*
Patition10	GTR+I+G	*rrnaL*
Patition11	GTR+I+G	*rrnaS*
